# Exploring the roles of information search and information evaluation literacy and pre-service teachers’ ICT self-efficacy in teaching

**DOI:** 10.1186/s41239-022-00339-5

**Published:** 2022-06-30

**Authors:** Palmira Peciuliauskiene, Giedre Tamoliune, Elena Trepule

**Affiliations:** 1 Education Academy, Vytautas Magnus University, T. Ševčenkos g. 31, 03111 Vilnius, Lithuania; 2 Education Academy, Vytautas Magnus University, V. Putvinskis str. 23-507, 44243 Kaunas, Lithuania; 3 Education Academy, Vytautas Magnus University, Jonavos str. 66-311, 44191 Kaunas, Lithuania

**Keywords:** Information search literacy, Information evaluation literacy, ICT self-efficacy in teaching, Pre-service teacher

## Abstract

This study analyzes the relationship between pre-service teachers’ information search and information evaluation literacy and their information and communication technology (ICT) self-efficacy in teaching. Theoretical analysis confirmed a direct relation between information literacy and pre-service teachers’ ICT self-efficacy in teaching. However, there is insufficient understanding regarding the effect that specific components of information literacy, i.e., information search and evaluation, have on teachers’ ICT self-efficacy in teaching. Data were collected using an online survey of students in teacher training programs who were engaged as research participants. The analysis disclosed that perceived information evaluation literacy has a more strongly expressed indirect impact on teachers’ ICT self-efficacy than a direct impact, while perceived information search literacy has a stronger direct impact on teachers’ ICT self-efficacy in teaching. Therefore, for teacher educators, it is important to develop a sufficient level of information literacy and ensure a positive experience with information search and evaluation, which are related to higher pre-service teachers’ ICT self-efficacy in teaching. This finding may help support calls for teacher training and/or professional development programs with a focus on information literacy, which might increase teachers’ ICT self-efficacy in teaching and improve ICT use in teaching.

## Introduction

The development of information and communication technology (ICT) has exploded at an impressive rate. In education, teachers are the key actors implementing and integrating ICT, as well as experts in teaching content (Ju Joo et al., 2018; Shonfeld et al., 2021). The successful integration of ICT in education depends on teachers’ personal readiness to use technological tools (Hatlevik & Hatlevik, 2018). Self-efficacy in teaching with ICT provides a foundation for personal comfort in the classroom.

According to Bandura (1994), individuals’ perceptions and beliefs regarding their thoughts and actions are perceived as self-efficacy. This means that self-efficacy beliefs determine individuals’ thoughts, feelings, and self-motivation. Christophersen et al., (2016, p. 2) stated that “teachers’ self-efficacy is about their beliefs and confidence of being capable of carry[ing] out good teaching in the classroom.”

Specific activities influence individuals’ self-efficacy. Krumsvik (2011) emphasized the importance of taking into account teachers’ self-efficacy, relating it to their teaching practice with ICT. Hatlevik and Hatlevik (2018) distinguished between general ICT self-efficacy and ICT self- efficacy for educational purposes. They stated that “general ICT self-efficacy is necessary for developing ICT self-efficacy for educational purposes and being able to use ICT in education” (p. 1). In this article, we analyze teachers’ self-efficacy in using ICT for teaching purposes—ICT self-efficacy in teaching.

ICT self-efficacy is directly related to teachers’ motivation to work (Barni et al., 2019), job satisfaction (Klassen & Chiu, 2010), the development of innovative learning designs, and engaging learners (Zee & Koomen, 2016) and contributes to overall well-being (Pinto, 2010; Zee & Koomen, 2016). Hammond et al. (2011) discovered a link between lower ICT self-efficacy and using ICT less frequently. Teo (2014) and Hatlevik (2017) revealed a positive association between self-efficacy regarding using digital tools and the use of ICT for teaching purposes. So et al. (2012) confirmed a positive relationship between pre-service teachers’ use of computers and their ICT self-efficacy.

Zee and Koomen (2016) noted that much research has been conducted over the last 40 years exploring the use of technologies in the classroom and teacher self-efficacy, disclosing that the use of ICT in teaching is directly related to teachers’ ICT self-efficacy. Research results (Karaseva, 2016) have confirmed that teachers’ self-efficacy depends on their information search literacy. However, even though teachers’ ICT self-efficacy for instructional use is related to their general ICT skills, there might be different levels of ICT self-efficacy, depending on whether it is related to ICT skills or to ICT use for instructional purposes (Hatlevik & Hatlevik, 2018).

Until recently, there was a lack of studies focusing on teachers’ ICT self-efficacy with regard to their information evaluation and information search literacy. Therefore, this paper aims to bring new knowledge to this field by disclosing the link between perceived information search and evaluation literacy, and teachers’ ICT self-efficacy for teaching purposes. The aim of this study is to explore the role of perceived information search and information evaluation literacy in pre-service teachers’ ICT self-efficacy in teaching. To this end, the following research questions were formulated:

- How does perceived information evaluation literacy predict pre-service teachers’ ICT self-efficacy in teaching?
- How does perceived information search literacy predict pre-service teachers’ ICT self-efficacy in teaching?
- How is the perceived information search literacy of pre-service teachers associated with their information evaluation literacy?
- What is the direct effect of perceived information evaluation literacy on pre-service teachers’ ICT self-efficacy in teaching?
- What is the indirect effect of perceived information evaluation literacy on pre-service teachers’ ICT self-efficacy in teaching?

## Theoretical background

### Information literacy

Initially, most information literacy research was conducted in the library research field, but, it has increasingly come into the scope of education research in the fields of higher and school education (Bundy, 2004; Johnston & Webber, 2005; Library Association, 2000; Secker & Coonan, 2013; Virkus, 2003). The changing nature of information resources requires changes in the curriculum (Eisenberg, 2010; Nevgi, 2007) in higher education, as well as teacher training. According to the Association of College and Research Libraries (Library Association, 2000, p. 1), “information literacy is a set of abilities requiring individuals to recognize the need for information and have the ability to locate, evaluate, and effectively use the information.” In a “new curriculum for information literacy” (Secker et al., 2011), student learning in the digital age depends on the information literacy that they hold as a set of skills, attributes, and behaviors.

The connection between information literacy research and education research in all sectors is strong (Bruce, 2016). Bhardwaj’s (2017) mapping of information literacy literature surveys in the humanities and social sciences for the period of 2001–2012 denoted rather intense research. Studies on teachers’ information literacy have revealed that insufficient information literacy may have an impact on how they transmit information literacy to learners (Durodolu, 2018). Shannon et al. (2019) argued that teachers have insufficient information literacy and noted that, in some cases, teachers were unfamiliar with the concept of information literacy and could not recognize information literacy in the content of their teaching curriculum. They also noted that not all teacher training courses include training in information literacy in the curriculum. These results complement other research results (Kohnen & Saul, 2018) disclosing that students do not develop adequate information search and evaluation literacy.

Information literacy includes a variety of abilities (Campbell, 2008), such as assessment of information quality and relevance to search goals, evaluating the reliability and timeliness of information, and applying new information to the creation and planning of professional pursuits. Pinto (2010) referred to the Association of College and Research Libraries (ACRL, 2010) Information Literacy Competency Standards for Higher Education, which contains five information literacy components:

(1) determine the nature and extent of the information needed, (2) search and accesses needed information effectively and efficiently, (3) evaluate information and its sources critically, (4) use information effectively to accomplish a specific purpose, (5) understand many of the economic, legal and social issues surrounding the use of information and accesses. (Library Association, 2000, p. 1)

In this article, we focus on two segments of information literacy: information search and information evaluation abilities.

Information search abilities, being part of information literacy (Campbell, 2008; Pinto, 2010) are closely related to ICT abilities. Even though students in teacher training usually belong to younger generations raised in environments where technology use was natural, research shows that digital nativity has a lower influence on information search literacy than on general information literacy (Çoklar et al., 2017). In other words, information search literacy does not automatically depend on learner digital nativity. Blummer and Kenton (2014) analyzed links between information search and the metacognition of students in education studies. In addition, new implications for information literacy have arisen in the environment of COVID-19 distance learning, and researchers are investigating the role of information search in teacher training (Tuluk & Akyuz, 2021), showing that, in some cases, teachers have sufficient knowledge of information search strategies.

Though it was initially considered that individuals could be information literate even without ICT literacy, in an information society, information literacy and ICT literacy are closely related (Catts & Lau, 2008). In the knowledge age of the twenty-first century, the ability to search for and access information by using digital tools is a critical one for teachers to transfer to the new generation. Head et al. (2020) argued that even though most undergraduate students have grown up with giants such as Google, YouTube, Instagram, or Facebook, they need information literacy to be confident users of digital media able not only to evaluate the information but also to foresee the algorithms that suggest content. In addition, research with 184 business CEOs in Finland has proven links between information literacy and innovation (Ahmad et al., 2019), indicating the connection of information literacy to the readiness to use innovative technologies.

Within information literacy, the category of information evaluation is related to a “student’s ability to analyze and manage information sources from any medium” (Pinto, 2010, p. 92). It is a student’s ability “to assess the quality of information by analyzing its usefulness, credibility, and relevance is related to their ability to use different sources and formats of information sources such as library catalogs, journals, databases, electronic books, and the internet” (Pinto, 2010, p. 92).

### ICT self-efficacy in teaching

The concept of self-efficacy derives from Bandura’s social-cognitive theory of behavioral change, where he defines self-efficacy as “people’s beliefs about their capabilities to produce designated levels of performance that exercise influence over events that affect their lives” (Bandura, 1994, 71). Self-efficacy can be context- or domain-specific, and Bandura suggested that, in most cases, it is more important to discuss domain-specific self-efficacy.

Gavora (2010) referred to teachers’ self-efficacy as “teacher’s personal belief in ability to plan instruction and accomplish instructional objectives” (p. 18). It is important to note that self-efficacy is not related to the multiple abilities that a teacher has but rather to belief regarding what a teacher can achieve with these abilities in a given situation (Bandura, 1994) and to the belief that tasks can be performed successfully (Cassidy & Eachus, 2002). Self-efficacy beliefs serve as a foundation for teachers’ motivation, personal accomplishments, and professional development. Since teachers’ self-efficacy was proven to have a positive impact on teachers’ motivation (Barni et al., 2019), to enable the development of a more innovative instructional design (Gavora), and to promote a more positive and responsive classroom environment (Alt, 2018), it is seen as a key to teachers’ actual teaching practice (Hatlevik, 2017).

Multiple research results have emphasized the connection between self-efficacy and educational outcomes (Zee & Koomen, 2016), and recent studies have contributed to this research field by disclosing how teacher self-efficacy is related to teaching quality (Buric & Kim, 2020). While a positive relation was found between teachers’ self-efficacy in using technological tools in general and using ICT in teaching practice (Hatlevik, 2017), and between the use of personal computers and prospective computer use (So et al., 2012), the frequency of ICT use appears to be rather important, since less frequent use of ICT is related to lower ICT self-efficacy (Hammond et al., 2011).

Hatlevik and Hatlevik (2018) noted that teachers’ ICT self-efficacy is dual, as it encompasses a general ICT self-efficacy and ICT self-efficacy for educational purposes. However, it is general ICT self-efficacy that supports and is important for ICT self-efficacy for instructional goals as well as ICT use in teaching practice. Moreover, teachers’ ICT self-efficacy is affected by both external and intrinsic factors, meaning that it can be affected by external factors such as school management (Hatlevik & Hatlevik, 2018; Holzberger & Prestele, 2021) or collegial collaboration (Hatlevik & Hatlevik, 2018). At the same time, ICT self-efficacy depends on such intrinsic factors as actual teaching experience (Hatlevik & Hatlevik, 2018), general use of ICT (So et al., 2012), frequency of using ICT (Hammond et al., 2011) or gender (Sabic et al., 2021). Moreover, it has been confirmed that the motivation to use and apply ICT in teaching practice increases with self-efficacy (Ju Joo et al., 2018; Krumsvik, 2014).

Teachers’ self-efficacy determines how much effort they expend on, e.g., information search and evaluation, and how resistant they are when handling difficulties and challenges in using or adapting ICT for teaching purposes. Hence, teachers’ success in using ICT in teaching depends on multiple factors, and information literacy appears to be a very important factor as well (Gavora, 2010). ICT teacher training can have a positive impact on and improve teachers’ self-efficacy in using computers; however, in some cases, being more capable in using ICT is not correlated with higher self-efficacy (Fanni et al., 2013).

### Establishment of null hypotheses

The development of information literacy is part of the teacher training curriculum. However, multiple studies have demonstrated that pre-service teachers do not seem to be prepared for new curriculums where ICT plays a major role (Gudmundsdottir & Hatlevik, 2020; Hatlevik, 2017). This relates to the importance of validating the trustworthiness of digital resources and information used for teaching purposes (Puustinen & Rouet, 2009). Since the role of teachers’ ICT self-efficacy in developing information evaluation strategies is substantial (Hatlevik), teachers working with online resources and information should be self-efficacious and know strategies for efficient information search and evaluation.

The previous theoretical analysis confirmed that information search and information evaluation are very important in educational practice; therefore, it is important to measure pre-service teachers’ perceptions about information search and information evaluation literacy (Campbell, 2008; Pinto, 2010; Tuluk & Akyuz, 2021). According to Pinto (2010), “information literacy (IL) is the set of literacies that an informed citizen needs in order to participate judiciously and actively in an information society” (p. 87). This set of literacies encompasses information search, evaluation, processing, and communication-dissemination (Pinto et al., 2019). Information literacy is related to the assessment of search results quality, evaluation of reliability, validity, as well as timeliness of information, application of new information to research, and other professional goals. Information search and evaluation includes abilities to find, access, and work with information sources from any medium. This literacy category includes learners’ ability to analyze and assess the quality of information by recognizing its timeliness and relevance (Pinto, 2010). The relationship between information search and information evaluation literacy confirms the main international standards and guidelines for information literacy, such as those of the American Library Association (ALA), the Association of College and Research Libraries (ACRL), and the Society of College, National and University Libraries (SCONUL) (SCONUL, 2011). They treat information search literacy and information evaluation literacy as internal aspects of the information literacy construct. In a holistic view treating information search and information evaluation literacy as internal components of information literacy, we hypothesized the following:

(**H**_**1**_) Perceived information evaluation literacy directly affects the information search literacy of pre-service teachers.

In this study, we focus on how two specific components of information literacy, i.e., information search and information evaluation literacy, affect teachers’ ICT self-efficacy in teaching. As discussed above, teachers’ information literacy encompasses a set of abilities that teachers should possess. In discussing information literacy, it is important to note that teachers with fewer information literacy skills are more likely to avoid handling information problem solving. By contrast, those who feel more confident in their information literacy skills will be more willing to tackle activities related to information problem solving (Kurbanoglu, 2003; Kurbanoglu et al., 2006). Pan and Franklin (2011) confirmed that positive prior experience with information search strategies is significantly related to the successful development of self-efficacy. This idea is supported by Karaseva (2016), who claimed that while teachers with low self-efficacy were least satisfied with their information search skills, as they struggled to choose relevant keywords, those with moderate internet self-efficacy felt rather satisfied with their search abilities—although they were dissatisfied with their information evaluation abilities. In addition, Pan and Franklin claimed that knowing information search strategies may result in teachers’ ICT self-efficacy. Thus, the second hypothesis we address is as follows:

(**H**_**2**_) Perceived information search literacy directly affects pre-service teachers' ICT self-efficacy in teaching.

With information search strategies, teachers are expected to develop information evaluation strategies. These are now critical for teachers, who may feel overwhelmed with the quantity of information available (Yan et al., 2016). According to Punie et al. (2013), evaluating information means being able to “gather, process, understand and critically evaluate information” (p. 5). Future teachers working with online information sources could benefit from well-developed strategies for information evaluation. They need to be able to critically assess and validate the trustworthiness of digital resources (Hatlevik, 2017; Puustinen & Rouet, 2009). Moreover, research has demonstrated a positive relationship between self-efficacy and information evaluation (Weinstein et al., 2000). ICT self-efficacy is necessary for future (and present) teachers, since they need to know strategies for critical, efficient information evaluation to find and select relevant and suitable resources for teaching and learning (Hatlevik, 2017). Hatlevik revealed that information evaluation strategies could be predicted by self-efficacy in basic ICT (β = 0.36, *p* < 0.01). Hatlevik argued that “more research is also required on how to develop teachers’ self-efficacy, their strategies to evaluate information, and their digital competence according to the competence aims in the curriculum” (p. 565). Considering this, the last two hypotheses we seek to address are as follows:

(**H**_**3**_) Perceived information evaluation literacy directly affects the ICT self-efficacy in teaching of pre-service teachers.

(**H**_**4**_) Perceived information evaluation literacy indirectly affects the ICT self-efficacy in teaching of pre-service teachers.

## Methods

### Data collection and research participants

The participants were students in teacher training programs at Vytautas Magnus University and Vilnius University in Lithuania. These universities are the largest teacher training centers in Lithuania.

The centers have different organizational models and didactical techniques involving (1) ICT across courses; (2) a separate ICT subject for the acquisition of ICT skills and/or pedagogical knowledge; (3) modeling or authentic planning, teaching, and evaluation of the use of ICT for learning; (4) practical use of ICT with children; and (5) online interactions with teacher professional communities and others.

Questionnaires were prepared and made available in online form. Invitations to participate in the study were distributed to the students. The survey was carried out online in May–June 2021 while universities were operating in online mode due to the COVID-19 situation. Since the survey was distributed online, participation was voluntary.

Three hundred and ten students were enrolled in the research, 200 (64.5%) from Vytautas Magnus University and 110 (35.5%) from Vilnius University. We enrolled more students from Vytautas Magnus University Education Academy because it is one of the largest teacher training centers in Lithuania. The Academy offers undergraduate study programs for future pre-school and primary school teachers, subject teachers, and special pedagogues for all school levels, as well as graduate programs for managers in education. The Academy also provides professional development and re-qualification courses for in-service teachers.

The representativeness of the study sample was ensured by simple random sampling. The reliability of the study sample was calculated using a 5% confidence interval and a 95% confidence level. According to the Education Management Information System of Lithuania, 1701 students studied in teacher training programs in 2021. This means that our chosen sample of 310 students was representative.

### Research model and instrument

In this study, pre-service teachers’ ICT self-efficacy in teaching, perceived information research skills, and information evaluation literacy were measured using a survey that was comprised of two validated questionnaires, one developed and validated by Markauskaite et al. (2006) and another by Pinto (2010). The use of both questionnaires was kindly approved by the authors, Lina Markauskaite and Maria Pinto, respectively.

**Fig. 1:**
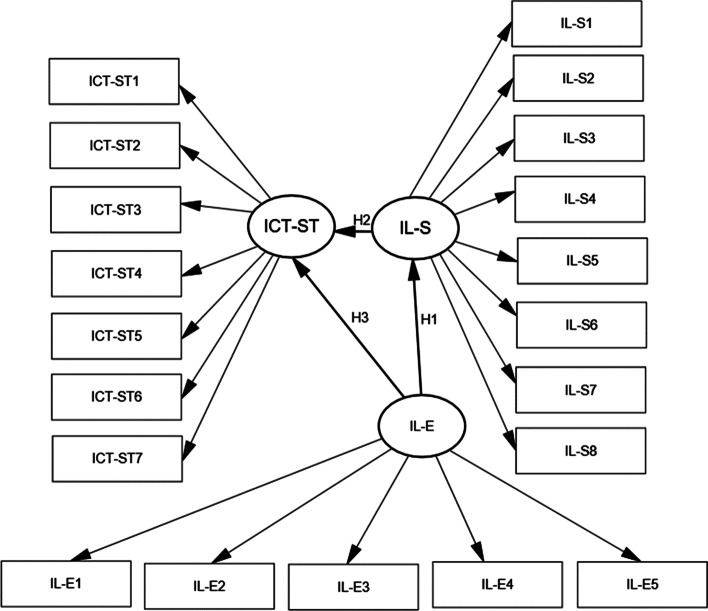
Structural model for structural equation modeling (SEM). Unobserved variables: information evaluation literacy (IL-E), information search literacy (IL-S), and pre-service teachers’ ICT self-efficacy in teaching (ICT-ST). Observed variables: (IL-E_n_); (IL-S_n_); (ICT-ST_n_)

For ICT self-efficacy in teaching, we used part of an ICT literacy self-assessment instrument developed by Markauskaite et al. (2006). This instrument is based on a dynamic model of ICT and measures pre-service teachers’ self-efficacy beliefs on their intention to use ICT in future work (“I believe that I will…”). Pre-service teachers’ beliefs about ICT use in their future careers encompass six components: enrichment of teaching and learning; communication and self-based learning; constructivist learning; teaching of general cognitive capabilities; teaching of ICT capabilities; and professional activities and development (Markauskaite et al., 2006). For the purposes of this study, we analyzed only one component—beliefs about ICT use for the enrichment of teaching and learning (seven items; Table 1). Based on this component, we created a latent variable (ICT-ST). In our model (Fig. 1), the latent variable (ICT-ST) is measured with seven observed variables. The unobserved variable (ICT-ST) is termed the latent factor.

**Table 1:** Internal content of latent variable: ICT self-efficacy in teaching (ICT-ST)

Code	Items: I believe that I will
ICT-ST_1_	Enrich classroom instruction with ICT activities
ICT-ST_2_	Use ICT in my regular classroom curriculum
ICT-ST_3_	Use multiple delivery methods for presenting new information
ICT-ST_4_	Design assignments in which students will need to submit work created using ICT
ICT-ST_5_	Design assignments in which students will need to do presentations with ICT tools
ICT-ST_6_	My students will use various mindtools software
ICT-ST_7_	Plan subject lessons in which students will learn ICT skills

We also used the validated instrument of Pinto (2010), a simplified version of Information Literacy Humanities Social Sciences (IL-HUMASS), for perception of information literacy. IL-HUMASS has been used in a number of studies (Pinto, 2010; Pinto et al., 2019, 2020, 2021). This instrument allowed us to explore the perceptions of pre-service teachers about the phenomenon of information literacy. According to IL-HUMASS, information literacy encompasses four categories: information search, information evaluation, information processing, and information dissemination. For the purposes of our study, we analyzed the perceptions of pre-service teachers about their information search (eight items) and information evaluation (five items) abilities. The responses helped us analyze two latent variables: perceived information search literacy (IL-S) and perceived information evaluation literacy (IL-E) (Table 2). In our model (Fig. 1), the latent factor (IL-S) is measured with eight observed variables, and the latent factor (IL-E) is measured with five observed variables (Table 2).

**Table 2:** Latent variables in perceived information literacy: information search literacy (IL-S) and information evaluation literacy (IL-E)

Latent variable	Code of observed variable	Observed variable
Information search literacy (IL-S)	IL-S_1_	To use printed sources of information (books, papers, etc.)
	IL-S_2_	To enter and use automated catalogues
	IL-S_3_	To consult and use electronic sources of primary information
	IL-S_4_	To use electronic sources of secondary information
	IL-S_5_	To know the terminology of your subject
	IL-S_6_	To search for and retrieve internet information
	IL-S_7_	To use informal electronic sources of information
	IL-S_8_	To know information search strategies
Information evaluation literacy (IL-E)	IL-E_1_	To assess the quality of information resources
	IL-E_2_	To recognize the author’s ideas within the text
	IL-E_3_	To know the typology of scientific information sources
	IL-E_4_	To determine whether an information resource is updated
	IL-E_5_	To know the most relevant authors within your subject area

We checked the convergent validity of the latent variables (ICT-ST), (IL-S), and (IL-E). We examined the convergent validity of latent variable (ICT-ST) by the average variance extracted (AVE) and composite reliability (CR); AVE = 0.523 > 0.50; CR = 0.910 > 0.70. Thus, the convergent validity and composite reliability of our latent construct (ICT-ST) are appropriate (Fornell & Larcker, 1981). We also analyzed the convergent validity of the latent construct information research literacy (IL-S) by the AVE and CR; AVE = 0.523 > 0.50; CR = 0.884 > 0.70. Therefore, the convergent validity and composite reliability are appropriate. Finally, we checked the convergent validity of the latent variable information evaluation literacy (IL-E) by the AVE and CR; AVE = 0.693 > 0.50; CR = 0.749 > 0.70. The convergent validity and composite reliability of IL-E are also appropriate.

We used Cronbach’s alpha to measure the internal consistency of the latent variable (ICT-ST). The results confirmed that the set of items is closely related as a group. Cronbach’s alpha for (ICT-ST) is equal to 0.883 > 0.65. We checked whether the (ICT-ST) data set corresponded to a model for a normal distribution by using skewness and excess kurtosis. The absolute values of skewness and kurtosis indicate the normality of the data set (i.e., absolute value is less than 2; Table 3).

**Table 3:** Pre-service teachers’ self-efficacy in enriching teaching and learning with ICT: normality of ICT-ST data set

	ICT-ST_1_	ICT-ST_2_	ICT-ST_3_	ICT-ST_4_	ICT-ST_5_	ICT-ST_6_	ICT-ST_7_
Skewness	− 1.028	− 1.231	− 1.758	− 0.921	− 0.860	− 1.040	− 1.042
*S.E*	0.152	0.152	0.152	0.152	0.152	0.152	0.152
Kurtosis	0.861	1.987	1.893	0.596	0.499	1.613	1.236
*S.E*	0.303	0.303	0.303	0.303	0.303	0.303	0.303

*S.E.* standard error

We used Cronbach’s alpha to measure the internal consistency of the latent variable (IL-S). The results confirmed that the set of (IL-S) items is closely related as a group. Cronbach’s alpha for (IL-S) is equal to 0.877 > 0.65. We checked whether the (IL-S) data set exhibited normal distribution. The absolute values of skewness and kurtosis indicate the normality of the (IL-S) data set (absolute values are less than 2; Table 4).

**Table 4:** Pre-service teachers’ perceived information search literacy: normality of (IL-S) model data

	IL-S_1_	IL-S_2_	IL-S_3_	IL-S_4_	IL-S_5_	IL-S_6_	IL-S_7_	IL-S_8_
Skewness	− 1.913	− 0.897	− 1.228	− 1.041	− 0.616	− 1.003	− 0.726	0.049
*S.E*	0.152	0.152	0.152	0.152	0.152	0.152	0.152	0.152
Kurtosis	1.317	0.310	1.213	0.693	0.562	0.474	− 0.416	− 0.420
*S.E*	0.303	0.303	0.303	0.303	0.303	0.303	0.303	0.303

*S.E.* standard error

The fitness of the latent variable (IL-E) items revealed sufficient fit and confirmed six questionnaire blocks. Cronbach’s alpha confirmed the internal reliability of this question group (0.877 > 0.650). We checked the normality of the (IL-E) data. The absolute values for skewness and kurtosis indicate the normality of the (IL-E) data set (absolute values are less than 2; Table 5).

**Table 5:** Pre-service teachers’ perceived information evaluation literacy (IL-E): normality of data

	IL-E_1_	IL-E_2_	IL-E_3_	IL-E_4_	IL-E_5_
Skewness	− 0.611	− 0.956	− 0.611	− 0.786	− 0.601
*S.E*	0.152	0.152	0.152	0.152	0.152
Kurtosis	0.336	2.051	0.116	0.590	0.598
*S.E*	0.303	0.303	0.303	0.303	0.303

*S.E.* standard error

## Results

We performed structural equation modeling (SEM) using the powerful SEM software Amos to test four hypotheses: (H_1_); (H_2_); (H_3_); (H_4_). SEM extends the possibility of relationships among the latent variables ([ICT-ST], [IL-S], and [IL-E]) and encompasses two components: a measurement model (essentially confirmatory factor analysis [CFA]) and a structural model (Fig. 1). Our model consists of exogenous (IL-E) and endogenous (ICT-ST; IL-S) variables (Fig. 1). Exogenous (IL-E) variable represents the construct that exert an influence on endogenous variables (IL-S; ICT-ST) in the structural model. Both exogenous and endogenous variables are unobserved (Fig. 1).

The internal structure of latent variables—pre-service teachers’ self-confidence in teaching (ICT-ST), perceived information search (IL-S) literacy, and perceived information evaluation (IL-E) literacy—was examined using CFA. We used goodness-of-fit indicators to assess the model: normed fit index (NFI), non-normed fit index (TLI), incremental fit index (IFI), comparative fit index (CFI), goodness-of-fit index (GFI), and root mean square error of approximation (RMSEA) (Table 6). The fitness of items for the latent variables revealed a sufficient fit and confirmed the questionnaire’s structure (Table 6).

**Table 6:** Fitness of items for the latent variables: Model ICT-ST, IL-S, IL-E, and final model

Model		Absolute fit index			Relative fit index		
		χ^2^/df	RMSEA	GFI	IFI	TLI	CFI
ICT-ST	Assumed model	2.007	0.063	0.979	0.990	0.976	0.990
IL-S	Assumed model	2.061	0.064	0.985	0.992	0.970	0.991
IL-E	Assumed model	1.271	0.033	0.994	0.998	0.993	0.998
Structural model	Assumed model	2.319	0.072	0.874	0.931	0.917	0.931
	Acceptance value	1–5	< 0.08	> 0.80	> 0.90	> 0.90	> 0.90

χ^2^ = absolute/predictive fit chi-square; *RMSEA*  root mean square error of approximation, *GFI*  goodness-of-fit index, *IFI*  incremental fit index, *TLI*  Tucker–Lewis index, *CFI*  comparative fit index

We performed CFA of the latent variable ICT self-efficacy in teaching (ICT-ST) (Fig. 1). Unstandardized coefficients (*B*) for observed variables and the latent factor (ICT-ST) were deducted (Table 7). The unstandardized beta value represents the influence of the predictor (observed) variable on the dependent (latent) variable. The highest unstandardized beta (*B*), for variable (ICT-ST_4_): this would mean that for everyone unit increase in variable (ICT-ST_4_), the dependent variable (ICT-ST) increases by 1.049 units (Table 7).

**Table 7:** Standardized and unstandardized coefficients of the latent variable ICT self-efficacy in teaching

Code of observed variable	Observed variable	*R*^*2*^	*B*	*S.E*	β	*p*
ICT-ST_1_	Enrich classroom *instruction* with ICT activities	0.515	0.835	0.077	0.718	<.001
ICT-ST_2_	Use ICT in my regular classroom *curriculum*	0.512	0.767	0.071	0.716	<.001
ICT-ST_3_	Use multiple delivery *methods* for presenting new information	0.427	0.673	0.067	0.654	<.001
ICT-ST_4_	Design *assignments* in which students will need to submit work using with ICT	0.498	0.948	0.087	0.706	<.001
ICT-ST_5_	Design *assignments* in which students will need to make *presentations* using ICT tools	0.584	1.049	0.090	0.764	<.001
ICT-ST_6_	Use various mindtools *software* for enhancing critical thinking and problem-solving skills	0.489	0.873	0.084	0.699	<.001
ICT-ST_7_	*Plan* subject *lessons* in which students will learn ICT skills	0.573	1.000		0.757	

*ICT-ST* ICT self-efficacy in teaching, *R*^*2*^  coefficient of determination, *B*  unstandardized coefficient, *S.E*.  standard error for unstandardized beta, *β*  standardized beta, *p*  probability level

Standardized beta (β) works very similarly to a correlation coefficient. Pre-service teachers’ self-efficacy in teaching with ICT has the strongest relationship with “design assignments—presentations using ICT tools” (β = 0.764; Table 4). The results of CFA revealed that the relation of the predictor (observed) variables to the dependent (latent) variable (ICT-ST) was statistically significant in all cases (Table 7).

The results of CFA by aspect of the coefficient of determination (*R*^*2*^) give the percentages of variations in the ICT-ST model explained by observed variables. The coefficient of determination (*R*^*2*^) value for the variable teachers’ ICT-ST differed by 42.7% to 58.4% (Table 4). This means that 42.7%–58.4% of the data fit the regression model.

We performed CFA of the latent variable “perceived information search literacy” (IL-S) (Tables 8, 9). CFA results revealed that the latent variable (IL-S) is statistically significantly related to all variables of perceived information search literacy (Table 8). The highest unstandardized beta values are for information search strategies (*B* = 1.731) and for searching for and retrieving internet information (*B* = 1.629), and lowest is for consulting and using electronic sources of primary information (*B* = 0.855; Table 6). The determination coefficient (*R*^*2*^) shows that the data are close to the regression lines because the absolute values of *R*^*2*^ are higher than 0.20 (Table 8).

**Table 8:** Standardized and unstandardized coefficients of latent variable pre-service teachers’ perceived information search literacy (IL-S)

Code of observed variable	Observed variable	*R*^*2*^	*B*	*S.E*	β	*p*
IL-S1	To use printed sources of information (books, papers, etc.)	0.279	1.000		0.528	
IL-S2	To enter and use automated catalogues	0.296	0.957	0.093	0.544	<.001
IL-S3	To consult and use electronic sources of primary information	0.260	0.855	0.111	0.510	<.001
IL-S4	To use electronic sources of secondary information	0.552	1.596	0.220	0.743	<.001
IL-S5	To know the terminology of your subject	0.566	1.579	0.212	0.752	<.001
IL-S6	To search for and retrieve internet information	0.626	1.629	0.212	0.791	<.001
IL-S7	To use informal electronic sources of information	0.358	1.264	0.196	0.598	<.001
IL-S8	To know information search strategies	0.566	1.731	0.243	0.753	<.001

*IL-S*  perceived information search literacy, *R*^*2*^ coefficient of determination, *B*  unstandardized coefficient, *S.E*.  standard error for unstandardized beta, *β*  standardized beta, *p* = probability level

**Table 9:** Standardized and unstandardized coefficients of latent variable teachers’ perceived information literacy: information evaluation abilities (IL-E)

Code of observed variable	Observed variable	*R*^*2*^	*B*	*S.E*	β	*p*
IL-E1	To assess the quality of information resources	0.283	0.791	0.114	0.532	<.001
IL-E2	To recognize the author’s ideas within the text	0.725	1.453	0.193	0.851	<.001
IL-E3	To know the typology of scientific information sources	0.765	1.456	0.194	0.875	<.001
IL-E4	To determine whether an information resource is updated	0.105	0.801	0.159	0.325	<.001
IL-E5	To know the most relevant authors within your subject area	0.234	1.000		0.484	

*IL-E*  perceived information evaluation literacy, *R*^*2*^  coefficient of determination, *B*  unstandardized coefficient,*S.E.*  standard error for unstandardized beta, *β*  standardized beta, *p*  probability level

We performed a CFA of the respondents’ perceived information evaluation according to IL-HUMAS, Pinto version (Pinto, 2010). Standardized and unstandardized coefficients for the observed variables and the latent variable (IL-E) were deducted (Table 9). The results revealed that pre-service teachers’ perceived recognition of the author’s ideas within the text was closely associated with pre-service teachers’ perceived information evaluation literacy (β = 0.851). The unstandardized beta was high (*B* = 1.453; Table 9). The CFA results also revealed that pre-service teachers’ perceived knowing the typology of scientific information sources was most associated with pre-service teachers’ perceived information evaluation literacy, with a high unstandardized beta value (*B* = 1.456).

We performed CFA by aspect of the coefficient of determination (*R*^*2*^). The coefficient of determination (*R*^*2*^) value for the latent variable (IL-E) was very high with the following: to recognize the author’s ideas within the text (0.725) and to know the typology of scientific information sources (0.765). This means that 76.5% of teachers’ perceived information evaluation literacy was affected by recognizing the author’s ideas within the text, and 72.5% by the knowing the typology of scientific information sources. It should be noted that the values of *R*^*2*^ are smaller than the factor to determine whether an information resource is up-to-date (*R*^*2*^ value is 0.105 < 0.20; Table 9).

The results of CFA revealed that the relation of the predictor (observed) variables to the dependent (latent) variable (IL-E) was statistically significant in all cases (Table 9).

As mentioned earlier, SEM, in comparison with CFA, extends the possibility of relationships among the latent variables. We tested the Structural Model (Fig. 1) and analyzed the relationship between perceived information evaluation literacy and perceived information search literacy (H_1_), the relationship between perceived information search literacy and ICT self-efficacy in teaching (H_2_), the relationship between the perceived information evaluation literacy and ICT self-efficacy in teaching (H_3_), and the indirect relationship between perceived information evaluation literacy and ICT self-efficacy in teaching (H_4_). The Structural Model provides the results drawing on maximum likelihood estimates (Table 6). It reports absolute fit measures.

We found that all direct and indirect paths were significant in the structural model (Table 10). The findings of SEM (*p* values) revealed that pre-service teachers’ perceived information evaluation literacy directly and positively affects perceived information search literacy (*B* = 0.993), (*R*^*2*^ = 0.533, *p* < 0.01). Perceived information evaluation literacy directly and positively affects ICT self-efficacy in teaching (*B* = 0.369), (*R*^*2*^ = 0.662, *p* < 0.01). It also revealed that perceived information search literacy directly and positively affects the ICT self-efficacy in the teaching of pre-service teachers (*B* = 0.553), (*R*^*2*^ = 0.662, *p* < 0.01). We also confirmed the hypothesis that perceived information evaluation literacy indirectly positively affects ICT self-efficacy in teaching (*B* = 0.424) (Table 10).

**Table 10:** Standardized and unstandardized coefficients of structural model: ICT self-efficacy in teaching; information searching; information evaluation literacy

Hypothesis	Path analysis	Effect	*R*^*2*^	*B*	*S.E*	β	*p*
H_1_ confirmed	IL-E → IL-S	Direct	0.533	0.993	0.107	0.730	<.001
H_2_ confirmed	IL-S → ICT-ST	Direct	0.662	0.553	0.096	0.581	<.001
H_3_ confirmed	IL-E → ICT-ST	Direct	0.662	0.369	0.110	0.285	<.001
H_4_ confirmed	IL-E → ICT-ST	Indirect		0.424		0.549	<.001

*ICT-ST*  ICT self-efficacy in teaching, *IL-S*  perceived information search literacy, *IL-E*  perceiving information evaluation literacy, *R*^*2*^  coefficient of determination,* B*  unstandardized coefficient, *S.E.*  standard error for unstandardized beta, *β*  standardized beta, *p*  probability level

The greatest *R*^*2*^ values involve H_2_ and H_3_ (Table 10). This means that 66.2% of pre-service teachers’ ICT self-efficacy in teaching is influenced by perceived information evaluation and search literacy. The remaining 33.8% is influenced by other factors.

## Discussion and conclusions

This study explores the role of perceived information search and information evaluation literacy on pre-service teachers’ ICT self-efficacy in teaching. Self-efficacy is defined as one’s belief in the ability to conduct a particular task successfully (Cassidy & Eachus, 2002). Related to one’s beliefs about the ability to perform specific tasks, self-efficacy regulates how one feels, thinks, self-motivates, and behaves when facing these tasks. ICT self-efficacy in teaching is defined here as pre-service teachers’ beliefs about their capabilities to use ICT in teaching. People who possess high self-efficacy view difficult tasks as challenges that can be mastered instead of seek to avoid them. Indeed, low self-efficacy may have a significant impact on one’s motivation and interest in examining information (Pinto, 2010). Hammond et al.’s (2011) study of the reasons teachers use ICT showed that “teachers with lower levels of self-efficacy in respect of ICT were among the least frequent users of ICT” (p. 196). Consequently, to improve the use of ICT in the educational process, one must consider ICT self-efficacy and the factors that determine it. On the basis of the theoretical background and by using SEM, we tested four hypotheses.

The first hypothesis aimed to test whether perceived information evaluation literacy directly affects the perceived information search literacy of pre-service teachers. Seeking to disclose the effect of information evaluation literacy on the information search literacy of pre-service teachers, we used the IL-HUMASS survey instrument. As mentioned, the IL-HUMASS is built on four questions related to the key categories of information literacy: searching, evaluation, processing, and communication and dissemination. Pinto et al. (2019) used the IL-HUMASS questionnaire to analyze the role of learning style (autonomous or directed learning) in the acquisition of information literacy competencies (searching, evaluation, processing, and communication-dissemination) among undergraduate social science students and revealed that students with a higher level of self-efficacy preferred directed learning to autonomous learning. The results demonstrated a higher preference for the directed learning style in all categories of information literacy competency, i.e., information search, evaluation, processing, and communication-dissemination, and these categories were considered components of a single construct (i.e., information literacy). We also treated information search and evaluation literacy as components of information literacy. In this study, we analyzed the associations among the perceived information search and evaluation literacy of pre-service teachers. We revealed that perceived information evaluation literacy is strongly associated with perceived information search literacy (Table 10).

The second and third hypotheses tested the direct effect of two information literacy components (perceived information evaluation literacy and perceived information search literacy) on the ICT self-efficacy in teaching of pre-service teachers. The results of hypothesis testing are in line with the results of other authors (Hatlevik, 2017; Kurbanoglu et al., 2006; Pan & Franklin, 2011). Hatlevik (2017) revealed that general ICT self-efficacy predicts information evaluation strategies. We therefore analyzed the inverse relationship between the ICT self-efficacy and information evaluation literacy of pre-service teachers. For the second hypothesis, analyzing the direct effects of perceived information evaluation literacy and perceived information search literacy on ICT self-efficacy in teaching, we noted that perceived information search literacy (IL-S) more reliably predicts ICT self-efficacy in teaching than perceived information evaluation literacy (IL-E) (Table 10). This finding seems to underpin the fact that having the ability to search for information allows one to feel more confident (Kurbanoglu et al., 2006) and, hence, perceive one’s self-efficacy. Positive experiences with information search strategies are directly related to how teachers perceive self-efficacy (Pan & Franklin, 2011).

The third hypothesis refers to perceived information search literacy directly affecting the ICT self-efficacy in teaching of pre-service teachers. A number of studies have been conducted analyzing information literacy self-efficacy (Tang & Tseng, 2013; Yan et al., 2016) . Yan et al. stated that information literacy self-efficacy is crucial in “coping with the negative effects of information overload in the modern information society” (p. 1098). In the context wherein an excess of information is available, information literacy allows us to differentiate “what information is needed, when it is needed, where it can be obtained, and how it can be effectively used” (Usluel, 2007, p. 92).

Analyzing the relationship between online learners’ self-efficacy and information literacy, Tang and Tseng (2013) revealed that self-efficacy for information searching is positively correlated with online learning self-efficacy, and online learning self-efficacy is positively correlated with information manipulation self-efficacy. Online learning is a form of ICT in education. We disclosed that information search literacy directly affects the ICT self-efficacy in teaching of pre-service teachers. In addition, analyzing the direct effects of perceived information evaluation literacy and perceived information search literacy of pre-service teachers on their ICT self-efficacy in teaching, we noticed that perceived information search literacy is a stronger predictor of ICT self-efficacy in teaching than perceived information evaluation literacy.

The fourth hypothesis is that perceived information evaluation literacy indirectly affects the ICT self-efficacy in teaching of pre-service teachers. Our study confirmed the associations between perceived information searching, information evaluation literacy, and ICT self-efficacy in teaching. The relationship between these constructs is direct and indirect (Table 10). Pinto (2010), analyzing the relationships among similar constructs, noted that low self-efficacy may be a significantly limiting factor for individuals exploring information (Pinto, 2010).

The results of hypothesis testing (Table 10) based on the structural model (Fig. 1) are in line with the results of other researchers (Fanni et al., 2013; Hatlevik, & Hatlevik, 2018; Klassen & Chiu, 2010; Krumsvik, 2011, 2014) who stated that teachers’ and pre-service teachers’ ICT self-efficacy could enable use ICT in their teaching practice and that higher ICT self-efficacy is related to higher confidence in using ICT for teaching purposes. Hammond et al. (2011) found that teachers with lower ICT self-efficacy used ICT less frequently. So et al. (2012) reported that ICT self-efficacy is related to both the use of personal computers and prospective computer use in teaching.

Our structural model (Fig. 1) has theoretical and practical significance. To refine the null hypotheses, we relied on an integral approach to the ICT self-efficacy and information literacy components—information search and evaluation literacy—of pre-service teachers. An integral approach to the research object and highlighting of direct and indirect pathways between information search and evaluation literacy and ICT self-efficacy constitutes the theoretical novelty of this study. On a theoretical level, the associations between information evaluation literacy and ICT self-efficacy, and the associations of information searching literacy and ICT self-efficacy of pre-service teachers have been updated by *SEM* results.

The results of *SEM* (Table 10) highlight practical issues for the information literacy study program developers of pre-service teachers both in terms of the organization of teaching and the principles of teaching. When developing information literacy curricula for teachers, it is important to allocate optimal time resources for the implementation of the study program. Based on the results of *SEM*, it is recommended to devote more time for the development of information search literacy of pre-service teachers than for the development their information evaluation literacy, as information search literacy directly more predict ICT self-efficacy of pre-service teachers (Table 10). In addition, *SEM* results suggest that information evaluation literacy not only directly but also indirectly predicts ICT self-efficacy of pre-service teachers (Table 10). Therefore, the principle of integration should be followed in the content development of information literacy study programs of pre-service teachers, both in terms of planning theoretical teaching materials and in terms of developing practical tasks.

Study program developers should consider that ICT used in educational practice are constantly evolving (Ju Joo et al., 2018; Shonfeld et al., 2021). Lack of information about new ICT reduces teachers’ ICT self-efficacy (Karaseva, 2016). However, as noted earlier, the implementation of new ICT in educational practice depends on ICT self-efficacy of teachers (Fanni et al., 2013; Hatlevik, & Hatlevik, 2018; Klassen & Chiu, 2010; Krumsvik, 2011, 2014). The results of *SEM* show that information search and evaluation literacy is an important factor in determining teachers’ ICT self-efficacy (Table 10). In pre- and in-service teacher training programs, information search and evaluation literacy (for teachers’ ICT self-efficacy) should be considered essential, as ICT self-efficacy has been confirmed to be closely related to ICT breakthrough in schools.

### Limitations

Two limitations in this paper’s research need to be mentioned. First, part of the study instrument was adapted from a questionnaire by Markauskaite et al. (2006) for ICT self-efficacy, when it related to ICT that was innovative at the time of the creation and validation of the instrument questionnaire. The pre-service teachers that participated in our survey may have treated ICT, as described in the questionnaire, not as innovation. The other limitation may be linked to the relatively small pre-service teacher population in Lithuania. Nevertheless, the required sample size was achieved, and the results are statistically valid. However, a similar survey of the ICT self-efficacy of pre-service teachers could be implemented in a different country, using a wider sample.

## Data Availability

The data sets generated and analyzed during the current study are not publicly available due to anonymity issues but are available from the corresponding author upon reasonable request.
